# Anti-Inflammatory and Antioxidant Effect of *Eucommia ulmoides* Polysaccharide in Hepatic Ischemia-Reperfusion Injury by Regulating ROS and the TLR-4-NF-*κ*B Pathway

**DOI:** 10.1155/2020/1860637

**Published:** 2020-05-25

**Authors:** Weidong Gao, Zanjie Feng, Shilong Zhang, Bo Wu, Xin Geng, Guoxin Fan, Yuling Duan, Kai Li, Kangwei Liu, Cijun Peng

**Affiliations:** ^1^Department of Hepatobiliary and Pancreatic Surgery, Affiliated Hospital of Zunyi Medical University, Zunyi, 563000 Guizhou, China; ^2^Department of Biochemistry and Molecular Biology, Zunyi Medical University, Zunyi, 563000 Guizhou, China

## Abstract

*Eucommia ulmoides* polysaccharide (EUP) has been shown to have anti-inflammatory and antioxidant effects. However, the mechanism underlying these effects has rarely been reported, and whether EUP can reduce liver injury in hepatic ischemia-reperfusion injury (HIRI) has not been reported. In this study, 40 Sprague-Dawley (SD) rats were randomly divided into 5 groups: the sham group, ischemia-reperfusion (I/R) group, and three EUP pretreatment groups (320 mg/kg, 160 mg/kg, and 80 mg/kg). SD rats were pretreated with EUP by gavage once a day prior to I/R injury for 10 days. Except for the sham group, blood flow in the middle and left liver lobes was blocked in all the other groups, resulting in 70% liver ischemia, and the ischemia and reperfusion times were 1 h and 4 h, respectively. Ischemic liver tissue and serum were obtained to detect biochemical markers and liver histopathological damage. Compared with the I/R group, after EUP pretreatment, serum alanine aminotransferase, aspartate aminotransferase, tumor necrosis factor-*α*, and interleukin-1*β* levels were significantly decreased, malondialdehyde levels in liver tissues were significantly decreased, superoxide dismutase levels were significantly increased, and the area of liver necrosis was notably reduced. To understand the specific mechanism involved, we determined the levels of Toll-like receptor- (TLR-) 4-nuclear factor-kappaB (NF-*κ*B) pathway-associated proteins in vivo and in vitro. The data showed that EUP can reduce liver damage by decreasing ROS levels and inhibiting TLR-4-NF-*κ*B pathway activation and may be a promising drug in liver surgery to prevent HIRI.

## 1. Introduction

Hepatic ischemia-reperfusion injury (HIRI), a phenomenon in which the structure and function of the ischemic liver are not restored after restoration of blood perfusion to the ischemic liver, but aggravated, is one of the main factors restricting the development of liver surgery. Swelling of endothelial cells and the Kupffer cells, neutrophil infiltration, and vasoconstriction are characteristic features of HIRI [1]. HIRI is common in liver surgery, especially hepatectomy, liver transplantation, and other surgical techniques. During major liver surgery, the blockage of hepatic blood flow is inevitable, and after recovery of the blood supply, ischemic liver necrosis or liver inactivation can occur, affecting patient recovery and even causing death [2–4]. Prevention of liver dysfunction caused by HIRI is an important strategy to improve the prognosis and survival of patients. Ischemia-reperfusion (I/R) injury is mainly caused by hypoxia and successive reoxygenation and usually occurs in oxygen-dependent organs, such as the heart, liver, lungs, and kidneys [5, 6]. An insufficient oxygen supply during hypoxia leads to adenosine triphosphate (ATP) synthesis disorders and abnormal cell metabolism, resulting in organ dysfunction [7]. The specific mechanism of HIRI is complicated and has not yet been fully elucidated; however, oxidative stress damage and aseptic inflammation play important roles in HIRI. In the early stage of HIRI, a large number of reactive oxygen species (ROS) including superoxide anions, hydrogen peroxide, hydroxyl radicals, and peroxynitrite are produced after the Kupffer cell activation, and the synthesis of inflammatory mediators such as tumor necrosis factor-*α* (TNF-*α*) and interleukin-1*β* (IL-1*β*) is increased, resulting in damage to liver cells [8, 9].

At present, research on HIRI prevention mainly includes ischemic preconditioning and drug preconditioning. Ischemic preconditioning has been proven to be effective in clinical practice, but its specific mechanism has not been clarified. The ischemic preconditioning time is critical, and it is restricted only to the treatment of acute liver injury, which limits its clinical application [10]. In recent years, more and more studies have been conducted on medicinal plants to treat HIRI, and many medicinal plant extracts and monomer components isolated from them have been confirmed to have therapeutic effects on HIRI, such as hypericum and curcumin [11, 12].

*Eucommia ulmoides* Oliver, known as du-zhong in China, is a dioecious woody plant endemic to China and is the sole species in the family Eucommiaceae [13]. *E. ulmoides* is widely cultivated in central and southern China and has been used as a herb to lower blood pressure and invigorate health effectively for at least 2,000 years. Using natural techniques, 204 natural compounds have been isolated and divided into seven categories, which have a wide range of pharmacological effects, such as antihypertensive, antioxidant, anti-inflammatory, and immunomodulatory effects [14].

*Eucommia ulmoides* polysaccharide (EUP) is a general term for the saccharides in *E. ulmoides* extracts. Polysaccharides consist of a wide range of biological macromolecules composed of the same or different monosaccharides and uric acid, are the basic components of all living objects, and are widely found in natural plants, microorganisms, and fungi [15, 16]. Polysaccharides in both simple and complex conjugated forms have a variety of functions in the body; existing studies have shown that EUP has anti-inflammatory, antioxidant, and immunoregulatory effects [17–20] and can alleviate renal ischemia-reperfusion injury in rabbits through antioxidant action [21]. Oxidative stress and the inflammatory response are important factors for promoting the development of HIRI [22], but whether EUP has a positive effect in HIRI has not been reported. Therefore, we conducted the following investigation to determine whether EUP has a protective effect in HIRI, and what type of pathway is adopted if there is a protective effect.

## 2. Materials and Methods

### 2.1. Materials

*E. ulmoides* polysaccharide (EUP, content: 60%, batch number TR20180607, extracted from *E. ulmoides* leaves) was obtained from Xi'an Tianrui Bio-Tech Co., Ltd. (Xi'an, China). The lipid peroxidation malondialdehyde (MDA) assay kit, dihydroethidium (DHE), and CCK-8 reagent were acquired from Beyotime (Shanghai, China), and the superoxide dismutase (SOD) assay kit was acquired from Nanjing Jiancheng Biological Engineering Institute (Nanjing, China). Primary antibodies against Toll-like receptor 4 (TLR4), high-mobility group protein B1 (HMGB1), myeloid differentiation factor 88 (MyD88), NF-*κ*B p65, IKB-*α*, interferon regulatory factor 1 (IRF-1), *β*-tubulin, *β*-actin, and horseradish peroxidase- (HRP-) conjugated secondary antibody were purchased from Proteintech (Wuhan, China). Primary antibodies against tumor necrosis factor-receptor-associated factor 6 (TRAF6), P-p65, and P-IKB-*α* were purchased from Affinity Biosciences (Cincinnati, OH, USA). HMGB1, TNF-*α*, and IL-1*β* ELISA kits were obtained from CUSABIO (Wuhan, China). Fetal bovine serum (FBS), RPMI-1640 medium, penicillin, and streptomycin were purchased from Gibco (Rockville, MD, USA). TLR-4 overexpression plasmid, empty plasmid, and polybrene were purchased from Hanbio Biotechnology Co., Ltd. (Shanghai, China).

### 2.2. Animals

Male Sprague-Dawley (SD) rats (180-220 g) were purchased from Changsha Tianqin Biotechnology Co., Ltd. All rats were raised under specific pathogen-free conditions with a 12 h day-night cycle. The rat experiments were performed in the Laboratory Animal Center of Zunyi Medical University, and all operations were conducted in accordance with the guidelines for the care and use of laboratory animals and were approved by the Local Institutional Committee of Zunyi Medical University of China, which agreed to choose the experimental animals for research (Approval No. ZMUER2016-2-054).

### 2.3. Liver I/R Model and Treatment

**Figure 1:**
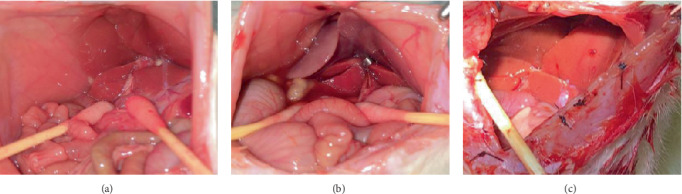
Liver changes in SD rats at different stages. (a) Nonligated liver vessels. (b) The liver vessels were ligated for 1 h. (c) After 4 h reperfusion. It can be seen from the images that after ligation for 1 h, the ischemic part of the liver was obviously dark, and the color of the liver returned to normal after blood flow was restored for 4 h, indicating that the HIRI model was successfully established.

The rats were randomly divided into five groups with 8 rats in each group: the sham group, I/R group, EUP high-dose group, EUP medium-dose group, and the EUP low-dose group. A 70% liver I/R model in rats was established in accordance with the method reported by Chi et al. [23]. In brief, rats were anesthetized with an intraperitoneal injection of pentobarbital sodium (60 mg/kg) before surgery, and an atraumatic clip was applied to the portal vessels to induce ischemia of the middle and left hepatic lobes. After 1 h ischemia, the clamp was removed for 4 h reperfusion, and the incision was closed (Figure 1). After 4 h reperfusion, blood samples were collected under anesthesia, and then the rats were killed by cervical dislocation immediately; then, parts of the I/R liver (middle and left lobes) were collected. In the EUP high-dose group, medium-dose group, and low-dose group, the rats were, respectively, treated with EUP at 320 mg/kg, 160 mg/kg, and 80 mg/kg by gavage for 10 days, once daily before I/R injury.

### 2.4. Cell Culture

Normal human hepatocyte cell line HL7702 (L02) was purchased from the Cell Bank of the Chinese Academy of Sciences (Shanghai, China). The cells were grown in RPMI-1640 medium containing 10% fetal bovine serum (FBS) and antibiotics consisted of 100 U/ml penicillin and 100 *μ*g/ml streptomycin. For incubation, all cells were housed at 37°C in a 5% CO_2_ incubator.

### 2.5. Cell Transfection

When the L02 cells in six-well plates grew to about 50% confluence, we replaced the medium with serum-free medium. The cells were then transfected with TLR-4 overexpression plasmid (NMID: NM_138554.1) or empty plasmid by lentivirus (MOI = 10) for 48 h, and polybrene is used to improve transfection efficiency (4 *μ*g/ml). The empty plasmid-transfected group was defined as the negative control (NC), and the TLR-4 overexpression plasmid group was defined as TLR-4.

### 2.6. Liver Function Assays

Serum alanine aminotransferase (ALT) and aspartate aminotransferase (AST) were detected by the Department of Clinical Laboratory, Affiliated Hospital of Zunyi Medical University, using an automatic biochemical analyzer.

### 2.7. Oxidative Stress Level Assays

The levels of MDA and SOD in liver tissues were detected using 10% liver homogenate according to the kit instructions. Frozen sections of 10 *μ*m were prepared from fresh liver tissue, ROS levels in liver tissue were detected by the DHE probe, and the nuclei were stained by DAPI.

### 2.8. ELISA

Rat serum was used to detect HMGB1, TNF-*α*, and IL-1*β* protein levels using the ELISA kit, according to the manufacturer's instructions.

### 2.9. H&E Staining of Liver Tissue

Liver tissue was fixed with 4% paraformaldehyde solution and then sent to the Department of Pathology of Zunyi Medical University for paraffin section and H&E staining.

### 2.10. Real-Time Quantitative PCR Assays

Total RNA was extracted from liver tissues using the RNAiso Plus reagent, and the total RNA concentration was quantified. Total RNA (500 ng) was reverse transcribed using the PrimeScript™ RT reagent kit (perfect real-time), and the cDNA obtained from reverse transcription was diluted 10 times with RNase-free distilled water. The TB Green™ Premix Ex Taq™ II (Tli RNaseH Plus) was used for quantitation. The following primers were used: HMGB1 (forward: 5′-ACAACACTGCTGCGGATGACAAG-3′; reverse: 5′-CCTCCTCGTCGTCTTCCTCTTCC-3′); TLR-4 (forward: 5′-GCTGCCAACATCATCCAGGAGG-3′; reverse: 5′-TGATGCCAGAGCGGCTACTCAG-3′); and beta-actin (forward: 5′-CACTATCGGCAATGAGCGGTTCC-3′; reverse: 5′-CAGCACTGTGTTGGCATAGAGGTC-3′).

### 2.11. Western Blot Assays

Total protein was extracted from liver tissues using RIPA cell lysis buffer containing protease inhibitors and phosphatase inhibitor. After the protein concentration was determined using the BCA assay, proteins were loaded on sodium dodecyl sulfate-polyacrylamide gels and transferred to a polyvinylidene fluoride membrane. After being blocked with 5% skimmed milk for 1 h (phosphorylated protein was incubated with 5% BSA), the membrane was incubated with primary antibodies at 4°C overnight. The membrane was incubated with HRP-conjugated secondary antibody at room temperature for 1 h after washing 3 times, for 10 min each time, then washed again using the same method. Chemiluminescent detection was performed using ECL reagents. The following primary antibodies were used: antibodies for HMGB1 (1 : 1000), TLR-4 (1 : 1000), MyD88 (1 : 1000), p65 (1 : 1000), P-p65 (1 : 1000), IKB-*α* (1 : 1000), P-IKB-*α* (1 : 1000), IRF-1 (1 : 1000), *β*-actin (1 : 5000), and *β*-tubulin (1 : 5000).

### 2.12. CCK-8 Analysis

In order to determine the appropriate concentration of EUP, CCK-8 assay was conducted to assess cell viability. Cells were maintained in 96-well plates (3 × 10^3^ cells/well), then incubated cells for 48 h with different concentrations of EUP the next day. Finally, 10 *μ*l/well of CCK-8 reagent was added. The absorbance was measured with a microplate reader at 450 nm.

### 2.13. Statistical Analysis

SPSS16.0 was used for analysis, and the results were expressed as mean ± standard deviation (mean ± SD). The one-way ANOVA was used to analyze differences between groups, and *P* < 0.05 was considered statistically significant.

## 3. Results

### 3.1. EUP Improves Liver Function following Hepatic Ischemia-Reperfusion Injury

**Figure 2:**
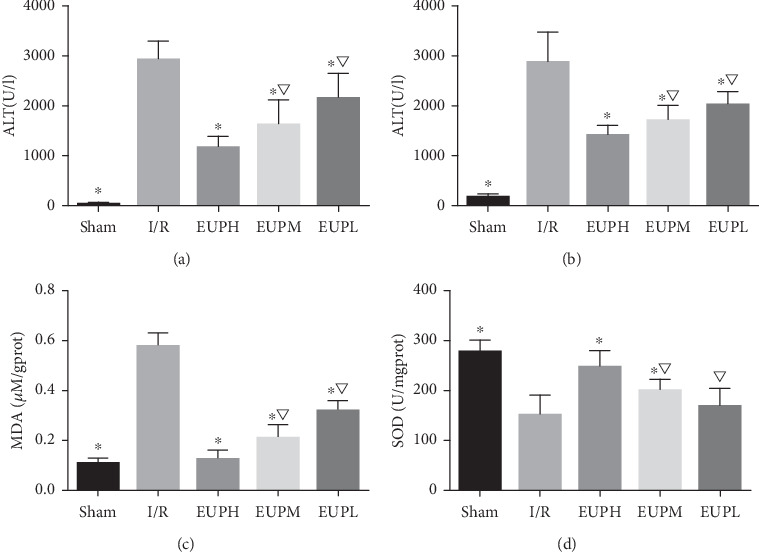
EUP improves liver function and oxidative stress injury in SD rats. Compared with the I/R group, ALT and AST were significantly decreased in the EUP pretreatment groups, and the reduction in the EUP high-dose (EUPH) pretreatment group was greater than that in the medium-dose (EUPM) and low-dose (EUPL) pretreatment groups (a, b). The difference was statistically significant. Similarly, in terms of reducing oxidative stress damage, compared with the I/R group, all of the EUP pretreatment groups showed significantly reduced oxidative stress damage caused by I/R, and the effect of EUPH pretreatment was most significant (c, d) (^★^compared with the I/R group, *P* < 0.05; ^▽^compared with the EUPH group, *P* < 0.05).

ALT and AST, two representative indices of liver function in serum, were measured. The results shown in Figures 2(a) and 2(b) demonstrate that ALT and AST levels in all the EUP pretreatment groups were significantly lower than those in the I/R group, with the EUP high-dose group showing the greatest reduction.

### 3.2. EUP Improved Oxidative Stress Injury Caused by I/R

MDA and SOD are key indicators of oxidative stress. MDA is an oxidation product and SOD represents the body's antioxidant capacity. As shown in Figures 2(c) and 2(d), EUP pretreatment significantly reduced the increase in MDA level caused by I/R, and the SOD level was significantly higher than that in the I/R group, with the EUP high-dose group showing the greatest reduction.

### 3.3. EUP Alleviated Inflammatory Response Injury Caused by I/R

**Figure 3:**
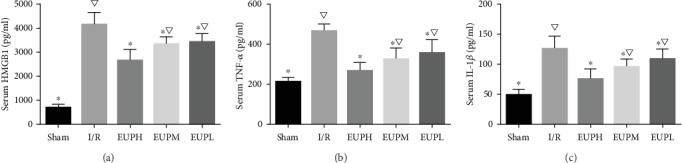
EUP reduces inflammatory cytokines in the blood. Rat serum was used to detect inflammation-related proteins (HMGB1, TNF-*α*, and IL-1*β*). Compared with the I/R group, EUP pretreatment significantly reduced the release of inflammatory factors, and EUP high-dose (EUPH) group had the greatest effect. The difference with statistically significance (^★^compared with the I/R group, *P* < 0.05. ^▽^compared with the EUPH group, *P* < 0.05).

HMGB1, as an important mediator for the sterile inflammatory response, was determined by ELISA. As shown in Figure 3(a), the content of HMGB1 in serum in the EUP pretreatment groups was significantly lower than that in the I/R group. In particular, the EUP high-dose group showed the greatest reduction. TNF-*α* and IL-1*β* are frequently tested indicators of inflammation and are often used to describe the level of inflammation in the body. EUP pretreatment significantly reduced the increase in TNF-*α* and IL-1*β* caused by I/R, and this reduction was greatest in the EUP high-dose group (Figures 3(b) and 3(c)).

### 3.4. EUP Alleviated Histological Injury of the Liver Caused by I/R

**Figure 4:**
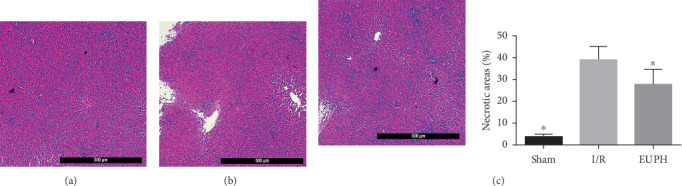
Effects of EUPH on histopathologic changes in the liver. As shown in the pathological sections from each group, compared to the sham group (a), large areas of hepatic necrosis were seen in the I/R group (b), and the necrotic areas were significantly reduced after pretreatment with high-dose EUP (c) (H&E staining, original magnification of 5 × 10, ^★^compared with the I/R group, *P* < 0.05).

The above results indicated that all three doses of EUP had protective effects in HIRI, and 320 mg/kg had the best protective effect; in order to further determine the protective effect of 320 mg/kg EUP on the liver, samples from the EUP high-dose group were used for liver hematoxylin and eosin (H&E) staining to observe the histopathologic changes. The results shown in Figure 4 demonstrate that liver injury was significantly reduced after treatment with 320 mg/kg EUP in rats compared with the I/R group.

### 3.5. EUP Downregulated the Increased Expression of Liver ROS Caused by I/R

**Figure 5:**
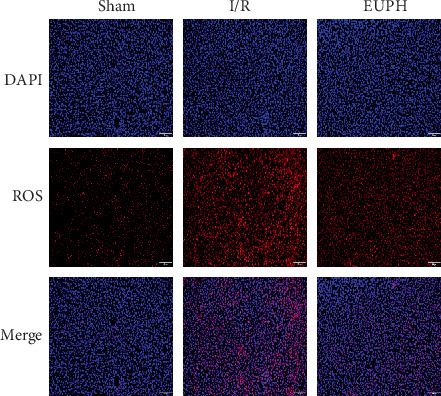
EUP attenuates the rise in ROS caused by I/R. ROS levels in liver frozen sections were detected using DHE probes. When the DHE probes were oxidized, they were combined with DNA and showed red fluorescence, and the nuclei were stained in blue with DAPI. Compared with the sham group, a large amount of red fluorescence was observed in the IR group, indicating that a large amount of ROS was produced in the liver when HIRI occurred, while a significant decrease in red substances was observed in the EUPH group. Staining results showed that pretreatment with 320 mg/kg EUP significantly reduced the rise in liver ROS caused by I/R (original magnification of 10 × 10).

Liver tissue was stained with the DHE probe to detect liver ROS content. The ROS levels in liver tissues in the EUP high-dose group were significantly decreased compared with the I/R group (Figure 5).

### 3.6. EUP Inhibited the TLR-4-NF-*κ*B Pathway after Liver I/R

**Figure 6:**
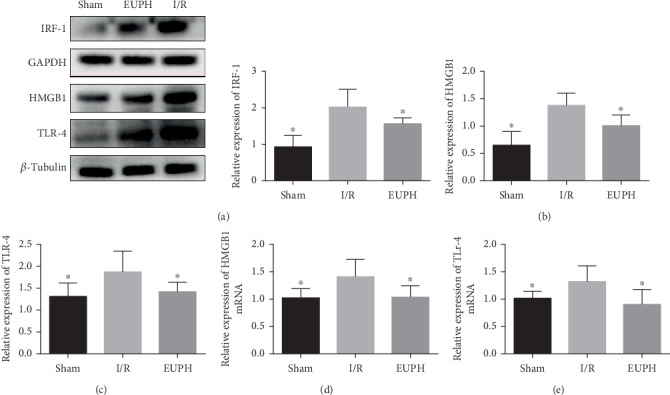
EUP alleviates the increased expression of IRF-1, HMGB1, and TLR-4 caused by liver I/R. IRF-1 (a), HMGB1 (b), and TLR-4 (c) proteins detected by Western blot, and both HMGB1 (d) and TLR-4 (e) mRNA were quantified by RT-PCR. Compared with the I/R group, the expression of IRF-1, HMGB1, and TLR-4 in the high-dose EUP group was significantly decreased. Data are presented as mean ± SD (^★^compared with the I/R group, *P* < 0.05).

**Figure 7:**
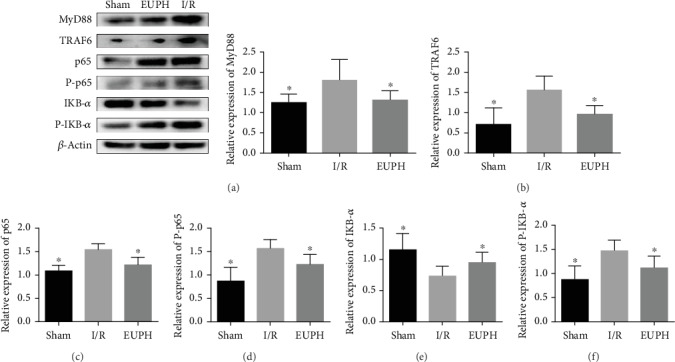
EUP inhibits activation of the NF-*κ*B pathway induced by liver I/R. Protein expression of MyD88 (a), TRAF6 (b), p65 (c), phospho-p65 (d), IKB-*α* (e), and phospho-IKB-*α* (f) were detected by Western blot, and the data are expressed as mean ± SD. Compared with the I/R group, the protein expression levels of MyD88, TRAF6, p65, phospho-p65, and phospho-IKB-*α* were significantly decreased in the EUP high-dose group, and the protein expression of IKB-*α* was significantly elevated in the EUP high-dose group. The results showed that EUP could significantly inhibit the phosphorylation of IKB-*α* and p65, thereby reducing the inflammatory response (^★^compared with the I/R group, *P* < 0.05).

As EUP pretreatment can significantly reduce the increase in HMGB1 release caused by I/R, in order to further explore the specific mechanism involved in the protective effect of EUPH in H/R, in this part, we detected HMGB1mRNA the downstream protein TLR-4 of HMGB1 and related proteins of the NF-*κ*B pathway, including HMGB1, TLR-4, MyD88, p65, P-p65, IKB-*α*, and P-IKB-*α* proteins, and TLR-4 mRNA. In addition, increased ROS expression can lead to increased synthesis of IRF-1, and IRF-1 can promote the separation of nonacetylated HMGB1 from DNA to acetylated HMGB1 and release it into the extracellular environment. Thus, we also measured the expression level of IRF-1 protein. EUP significantly inhibited the increased expression of IRF-1, HMGB1, and TLR-4 caused by I/R (Figure 6), as well as activation of the downstream TLR-4-NF-*κ*B pathway (Figure 7).

### 3.7. EUP Promoted L02 Cells Proliferation

**Figure 8:**
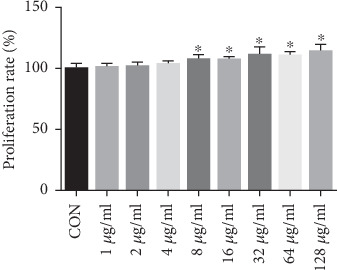
EUP promoted L02 cells proliferation. Effects of different concentrations of EUP on L02 cells were detected by CCK-8, and the data are expressed as mean ± SD. Compared with the CON group, EUP concentration from 8 *μ*g/ml to 128 *μ*g/ml has the effect of promoting proliferation of L02 cells, and as the concentration increases, the promoting effect is more obvious (^★^compared with the CON group, *P* < 0.05).

In order to further observe the inhibitory effect of EUP on TLR-4, an in vitro experiment was designed. We used different concentrations of EUP to incubate L02 cells for 48 h to observe the effect of EUP on cells. The results show that EUP concentration of 8 *μ*g/ml and above promoted the proliferation of L02 cells (Figure 8).

### 3.8. EUP Inhibited the TLR-4-NF-*κ*B Pathway In Vitro

**Figure 9:**
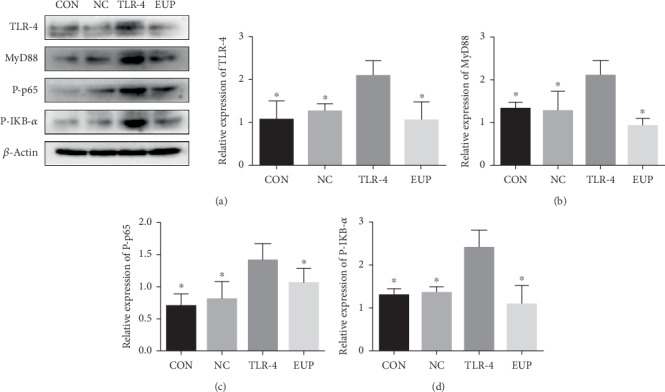
EUP inhibited the TLR-4-NF-*κ*B pathway in vitro. Protein expression of TLR-4 (a), MyD88 (b), P-p65 (c), and P-IKB-*α* (d) were detected by Western blot, and the data are expressed as mean ± SD. After TLR-4 was overexpressed in L02 cells, the TLR-4-NF-*κ*B pathway was obviously activated, which resulted in increased MyD88 protein expression and promoted phosphorylation of p65 and IKB-*α*. However, TLP-4 overexpressing cells treated with EUP obviously inhibited the activation of TLR-4-NF-*κ*B pathway (^★^compared with the TLR-4 group, *P* < 0.05).

Based on the results of the CCK-8 experiment, we finally determined to use 8 *μ*g/ml EUP to observe the inhibitory effect on the TLR-4/NF-*κ*B pathway in vitro. In this part, we set up 4 groups, namely the CON group, NC group, TLR-4 group, and EUP group (EUP treatment of TLR-4 overexpressing L02 cells). Cells of different groups were seeded in 6-well plates. When the confluence of cells reached 80%, the medium was changed. The medium in the EUP group contained 8 *μ*g/ml EUP. After 48 hours, the expression levels of TLR-4, MyD88, P-p65, and P-IKB-*α* proteins in each group were detected. EUP significantly inhibited the increased expression of TLR-4, MyD88, P-p65, and P-IKB-*α* proteins (Figure 9).

## 4. Discussion

Liver surgery is an effective treatment for benign or malignant tumors as well as end-stage liver disease, but the accompanying HIRI has a negative impact on postoperative recovery and even causes death. Preventing the occurrence and development of HIRI is a major challenge in liver surgery. Previous studies have found that HIRI can be classified into two phases. Firstly, intracellular oxygen levels decrease with ischemia, causing ATP synthesis disorders and cellular metabolic disorders, while ROS, cytokines, and adhesion molecules increased significantly, causing further damage [24, 25]. Secondly, the damage caused by reperfusion is also the main period of liver damage and failure. Following the restoration of liver blood flow, the ischemic liver changes from metabolic distress to an excessive innate immune response, and a large number of neutrophils penetrate the reperfused liver, which is the main cause of cell damage during reperfusion [26].

Under normal circumstances, the ability of liver cells to produce ROS and the efficiency of SOD to scavenge ROS are in a state of dynamic equilibrium, known as ROS homeostasis [27]. Low concentrations of ROS are essential for normal physiological activity of cells, but when ROS increases, it destroys lipids and causes damage to cells [28]. Mitochondrial respiratory chain complexes, the main sites of ROS production, transfer electrons to oxygen. Due to the incomplete reduction of oxygen, superoxide radicals are produced as by-products of the process, in which complexes I and III are considered to be the main source of mitochondrial superoxide radicals. Superoxide radicals can be converted to hydrogen peroxide by mitochondrial SOD, which is then cleared by catalase [29–32]. When liver ischemia occurs, the oxygen source is limited and mitochondria produce a large amount of ROS, which is accompanied by a decrease in SOD level. The increased ROS attacks lipids and produces a large amount of MDA. Our study showed that EUP can reduce oxidative stress damage by reducing the increase in ROS caused by I/R, increasing SOD level and reducing the production of lipid metabolism end product (MDA).

In the early stage of reperfusion injury, damage-associated molecular patterns (DAMPs) initiate and maintain subsequent complex aseptic inflammatory reactions, creating local conditions for the recruitment of neutrophils [33, 34]. DAMPs are immunomodulatory intracellular molecules released after cell damage or activation, including HMGB1, heat shock proteins, nonprotein purine molecules, and their degradation products and degradation products of extracellular matrix [35]. HMGB1, one of the most studied DAMPs in HIRI, is a highly conserved DNA-binding protein consisting of 215 amino acids widely distributed in eukaryotic cells [36]. HMGB1 in different parts has different functions; HMGB1 exists in the nucleus in a deacetylated form and has functions such as stabilizing nucleosome structure, regulating transcription factors, and participating in various biological processes such as gene transcription and DNA repair. Intracellular HMGB1 is involved in autophagy and vesicle formation, and extracellular HMGB1 is considered to be an important mediator of inflammation after cell damage or infection [37, 38]. In HIRI, HMGB1 began to be released at the early stage of reperfusion. The experiment of Tsung et al. showed that HMGB1 began to rise in the liver of rats after 1-hour reperfusion and then increased in a time-dependent manner up to 24 h. Neutralizing antibodies used before I/R to inhibit the activity of HMGB1 can significantly reduce the liver injury in rats, which proves that HMGB1 is a relatively sensitive indicator of HIRI and mediates the liver injury after IR [39]. In our experiment, HMGB1 was significantly increased after 1 h of ischemia and 4 h of reperfusion in rats, and HMGB1 content in liver tissues was significantly decreased after pretreatment with EUP, accompanied by the decrease of ALT and AST, as well as the decrease of liver necrotic areas. In addition, HMGB1 secreted into serum can be expanded to the tissues throughout the body with blood circulation, causing damage to multiple organs throughout the body. In our experiments, it was found that EUP can significantly reduce the HMGB1 content in serum, thereby alleviating other organ damage caused by HIRI.

A large amount of ROS produced by cell damage promotes the expression of early response IRF-1 and elevates cytosolic Ca^2+^ levels. IRF-1 enhances histone acetyltransferase (HAT) activity and promotes acetylation of HMGB1, and acetylated HMGB1 detaches from DNA and enters the cytoplasm from the nucleus. Cytoplasmic Ca^2+^ further promotes the combination of HMGB1 into secretory lysosomes, which are then secreted outside the cell to mediate the inflammatory response [40, 41]. Extracellular HMGB1 binds to the TLR-4 receptor and activates the NF-*κ*B pathway.

Since Buetler et al. first reported that TLR-4 is critical for the identification of bacterial lipopolysaccharides, TLR-4 receptors have been extensively studied in inflammatory response. Subsequent studies have found that TLR-4 also plays a key role in the activation of sterile inflammatory responses [42]. TLR-4 mediates inflammation through two different pathways—myeloid differentiation factor 88- (MyD88-) dependent pathway and MyD88-independent pathway. In MyD88-dependent pathway, the activation of TLR-4 increases the activation of its downstream MyD88; then, IL-1 receptor-associated kinase and tumor necrosis factor-receptor-associated factor 6 (TRAF6) are recruited to mediate the activation of the inhibitor of KB (IKB) complex (IKK) and the degradation of IKB-*α* [43]. NF-*κ*B and IKB-*α* form a polymer, when cells are stimulated by inflammation, and IKB-*α* is degraded by phosphorylation. NF-*κ*B phosphorylation plays a proinflammatory role, leading to the production of a large number of inflammatory mediators such as TNF-*α* and IL-1*β*, resulting in hepatocyte damage. In the present study, pretreatment with EUP significantly reduced TLR-4 protein and mRNA expression, reduced IRF-1 protein expression, inhibited NF-*κ*B activation, and reduced serum TNF-*α* and IL-1*β* levels. To further confirm the inhibitory effect of EUP on the TLR-4-NF-*κ*B pathway, we designed an in vitro experiment. The plasmid was used to overexpress TLR-4 protein of L02 cells to mimic the activation of the TLR-4-NF-*κ*B pathway in HIRI. The experimental results show that EUP could promoted proliferation of L02 cells and significantly inhibit the increased expression of TLR-4, MyD88, P-p65, and P-IKB-*α* proteins. These findings suggest that EUP can attenuate the inflammatory response caused by the activation of the TLR-4-NF-*κ*B pathway and reduce the sterile inflammatory response in the liver.

**Figure 10:**
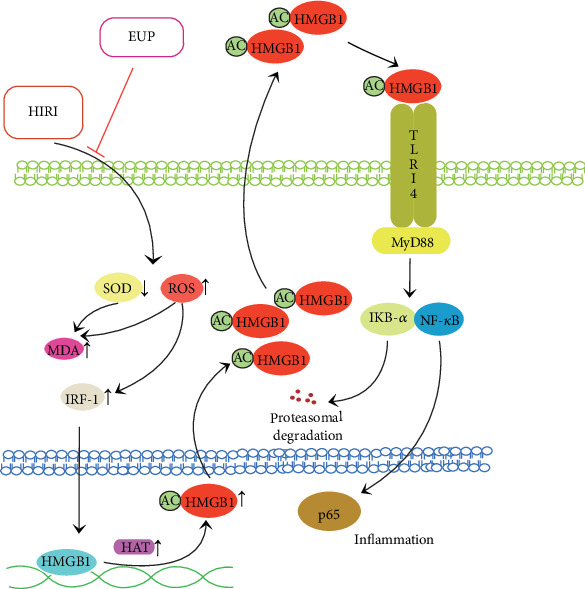
The role of EUP in hepatic ischemia-reperfusion injury.

Plant extracts have the advantages of a wide range of sources, and there are more and more researches in various fields such as anti-inflammatory and antioxidation. In addition, because of their relatively low cost, they are more suitable for patient acceptance and have broad development prospects. Previous reports have confirmed that EUP has antioxidant and anti-inflammatory effects, but research on EUP is still rare. This study showed that EUP can play an anti-inflammatory and antioxidant role by lowering ROS levels, increasing SOD levels, and inhibiting TLR-4-NF-*κ*B pathway activation by reducing HMGB1 release inhibition (Figure 10). The combination of the two pathways ultimately reduces the degree of liver necrosis and reduces liver function damage.

## 5. Conclusion

EUP pretreatment in the I/R model reduced oxidative stress damage and inhibited activation of the TLR-4-NF-*κ*B pathway by reducing ROS release and finally reducing liver damage.

## Data Availability

The datasets used and/or analyzed in this study are available from the corresponding authors on reasonable request.
